# Diffuse Reflectance Spectroscopy as a Novel Method of Caries Detection—An In Vitro Comparative Study in Permanent Teeth

**DOI:** 10.3390/diagnostics13111878

**Published:** 2023-05-27

**Authors:** Jindrich Charvat, Ales Prochazka, Tomas Kucera, Antonin Tichy, Maksim Yurchenko, Lucie Himmlova

**Affiliations:** 1 Institute of Dental Medicine, First Faculty of Medicine of the Charles University and General University Hospital in Prague, 121 08 Prague, Czech Republic; jindrich.charvat2@lf1.cuni.cz (J.C.); antonin.tichy@lf1.cuni.cz (A.T.);; 2 Department of Computing and Control Engineering, University of Chemistry and Technology in Prague, 166 28 Prague, Czech Republic; ales.prochazka@vscht.cz; 3 Czech Institute of Informatics, Robotics and Cybernetics, Czech Technical University in Prague, 166 36 Prague, Czech Republic; 4 Institute of Histology and Embryology, First Faculty of Medicine, Charles University in Prague, 128 01 Prague, Czech Republic; tomas.kucera@lf1.cuni.cz

**Keywords:** caries detection, diffuse reflectance spectroscopy, diagnostic, histology, laser fluorescence, Diagnodent pen

## Abstract

This in vitro study aimed to compare outcomes of dental caries detection using visual inspection classified according to the International Caries Detection and Assessment System (ICDAS) with objective assessments using a well-established laser fluorescence system (Diagnodent pen) and a novel diffuse reflectance spectroscopy (DRS) device. One hundred extracted permanent premolars and molars were utilized, including sound teeth, teeth with non-cavitated caries, or teeth with small cavitated lesions. A total of 300 regions of interest (ROIs) were assessed using each detection method. Visual inspection, being a subjective method, was performed by two independent examiners. The presence and extent of caries were histologically verified according to Downer’s criteria, serving as a reference for other detection methods. Histological results revealed 180 sound ROIs and 120 carious ROIs, categorized into three different extents of caries. Overall, there was no significant difference between the detection methods in sensitivity (0.90–0.93) and false negative rate (0.05–0.07). However, DRS exhibited superior performance in specificity (0.98), accuracy (0.95), and false positive rate (0.04) compared to other detection methods. Although the tested DRS prototype device exhibited limited penetration depth, it shows promise as a method, particularly for the detection of incipient caries.

## 1. Introduction

Despite well-known preventive measures, dental caries is still among the most prevalent infectious diseases in the world. While the focus should be placed on primary prevention, early detection of caries is essential to prevent irreversible damage to enamel and dentin that would subsequently lead to the need for an invasive intervention [1,2]. Currently, caries diagnosis still relies predominantly on the subjective assessment of the examining dentist, as visual inspection is usually combined with radiographic examination [3]. The accuracy of visual inspection can be improved by using detailed and validated indices [4], such as the International Caries Detection and Assessment System (ICDAS, Table 1) that distinguishes various stages of caries [5,6]. A recent systematic review concluded that the ICDAS presented a substantial level of reproducibility and accuracy for assessing primary coronal caries lesions [7]. Nevertheless, the outcomes of visual inspection are also strongly influenced by the experience and knowledge of the examining clinician [4,8,9,10].

To improve the accuracy of caries detection, various non-invasive complementary methods have been introduced. Besides X-rays, these methods utilize visible light (fiber optic transillumination, quantitative light-induced fluorescence), laser fluorescence, optical coherence tomography, ultrasound, or electrical current (conductance and impedance measurements) [11,12]. However, their clinical application is relatively scarce, except for digital fiber optic transillumination (DIFOTI) and laser fluorescence. DIFOTI employs a source of intensive light that visualizes caries and/or cracks of individual teeth and a digital camera that captures images of the transilluminated tooth structures. The commercially available device—DIAGNOcam (KaVo Dental, Biberach an der Riss, Germany)—uses a laser diode emitting light that has a wavelength of 780 nm, and previous studies proved that its ability to detect proximal caries is comparable to radiographic assessment and superior to visual examination [13,14,15]. The main drawbacks of DIFOTI include its inability to differentiate between carious lesions and developmental defects and its lack of objectivity, as the image is subjectively analyzed by the clinician [12].

An objective scale is available for a commercial device based on laser fluorescence—the Diagnodent pen (KaVo Dental, Biberach an der Riss, Germany). A low-intensity laser with a wavelength of 655 nm interacts with organic molecules, such as protoporphyrin IX and related metabolic products of cariogenic bacteria, resulting in fluorescence. The device detects the fluorescent light and displays a numerical value (scale 0–99) that expresses the condition of the tooth [16]. This device exhibited remarkable results in the detection of occlusal caries [17,18,19,20,21,22,23,24,25]. Nevertheless, it has not gained much acceptance among dental practitioners because the assessment is more time-consuming than visual examination, it requires a different tip for the detection of proximal caries, and its outcome is affected by the presence of saliva, stain, calculus, and plaque [13,26,27,28,29].

Spectroscopic methods have been tested for caries detection as well in the last decades [30,31,32,33,34]. The most recently introduced diffuse reflectance spectroscopy (DRS) analyzes light backscattered from the inside of a studied object/tissue rather than light reflected from its surface [35,36,37]. Furthermore, DRS uses a broad range of wavelengths in the visible and near-infrared spectrum, as opposed to the narrow spectrum of lasers used as a source of light in previous applications of spectroscopy for caries detection. As a result, DRS has been able to show the loss of light reflection in the blue–green region of the spectrum (<570 nm) [38] and increased reflectance at longer wavelengths (particularly 800–1000 nm) in carious tissues, which have a decreased content of minerals and increased content of water [37].

To date, DRS has only been tested in vitro but it exhibited promising results. Depending on the parameters of the device and the selected method of data analysis, DRS achieved accuracy ranging from 84.0% to 98.4% [35,36,37]. However, to our knowledge, DRS has not been compared to other clinical caries detection methods used in contemporary dentistry. Therefore, the aim of this study was to evaluate the ability of DRS to detect dental caries in extracted human permanent teeth and to compare it with visual inspection (ICDAS) and laser fluorescence (Diagnodent pen). Histological analysis of the teeth was performed to verify the presence of caries and to classify their extent. The histological results then served as a reference for each of the evaluated detection methods. The null hypotheses were that there would be no difference (1) between the detection methods and (2) between their accuracy in detecting different stages of caries.

## 2. Materials and Methods

The institutional ethical committee approved the use of extracted human teeth in this study (protocol number 613/18 S-IV). The teeth were extracted for periodontal, orthodontic, prosthetic, or surgical reasons after obtaining each patient’s written informed consent for the extraction and the use of the teeth for research purposes. After extraction, the teeth were debrided, stored at 4 °C in a 0.5% chloramine-T solution for 1 week, and then transferred to distilled water, which was weekly changed. Prior to assessments, the teeth were cleaned using a rotary brush with an abrasive paste (Depural Neo; SpofaDental; Jicin, Czech Republic) at 10,000 rpm and thoroughly rinsed with water. For the experiments, the principal investigator (J.C.) selected 100 permanent teeth (premolars and molars) that were either sound or presented caries corresponding to ICDAS codes 1–3 (Table 1). The principal investigator also determined 3 regions of interest (ROIs) per tooth with respect to the planned sectioning for histological analysis. ROIs were selected in areas susceptible to caries, i.e., in pits and fissures of occlusal surfaces (*n* = 165), and in discolored cervical areas (*n* = 135). All ROIs were photographically recorded using a digital camera (Nikon D5100; Nikon, Tokyo, Japan) with a 90 mm macro lens (F/2.8) to ensure that the same location was assessed using all the methods.

### 2.1. Visual Inspection

The ROIs were independently assessed by two dentists who received training in ICDAS—L.H. (30 years since graduation) and J.C. (7 years since graduation). The assessments were performed under a standard dental light with bare eyes (without magnification). The dentists used a three-way dental syringe to dry the ROIs for 5 s, and the assessment was only visual, no probe was used. [5,6,7].

### 2.2. Laser Fluorescence (Diagnodent Pen) Assessment

A laser fluorescence device (Diagnodent pen IR 2190; KaVo Dental, Biberach an der Riss, Germany) was used for the assessment. The principal examiner (J.C.) calibrated the probe (Diagnodent pen sapphire fissure probe) prior to the assessment using a ceramic standard supplied by the manufacturer, air-dried the ROIs for 5 s, and evaluated them by rotational movements of the attached probe. The highest detected value was recorded [39,40], and the obtained results were interpreted in accordance with the manufacturer’s scale (Table 2).

### 2.3. Diffuse Reflectance Spectroscopy (DRS) Assessment

**Figure 1:**
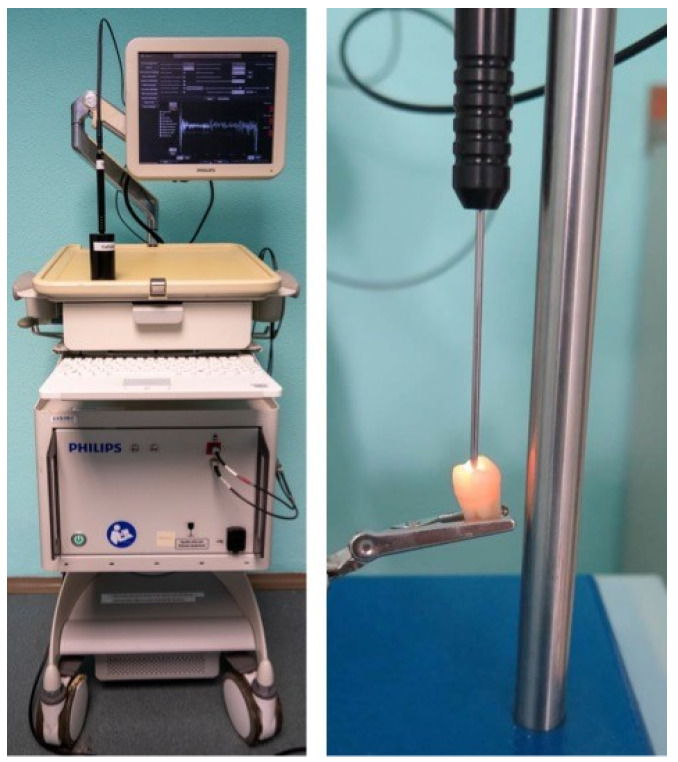
The DRS device (on the **left**) and a detail of the probe attached to a tooth (on the **right**).

A prototype of a DRS device, developed by Philips Research (Eindhoven, The Netherlands), was used for the assessment (Figure 1). The device consists of a halogen broadband light source (400–1600 nm) and a portable probe with two optical fibers of mutual distance of 0.85 mm. The optical fibers are connected to an InGaAs spectrometer with Horiba-S330-2 and Horiba-S318-2 VIS detectors (Horiba, Kyoto, Japan) measuring in the range of 10–984 nm and 845–1730 nm, respectively. 

The assessments were performed by the principal investigator (J.C.) who was trained by the staff of Philips Research. Prior to each series of measurements, calibration was performed using a white standard (Spectralon White Diffuse Reflectance Standard (99%), Edmund Optics, Barrington, NJ, USA). The setup of the calibrated device was checked regularly by measuring a reference tooth at a given location to ensure the repeatability of the measurements throughout the whole study.

Each ROI was air-dried for 5 s, measured ten times, and median reflectivity values at each wavelength were analyzed using previously developed algorithms [42,43]. The following features were used for the classification of ROIs:

1. Wavelength at which reflectivity was maximal (λ_max_);
2. Wavelength at which reflectivity was minimal (λ_min_);
3. Average reflectivity in the interval of wavelengths λ_max_—λ_min_;
4. Average difference between reflectivity values in the λ_max_—λ_min_ interval and a line connecting maximal and minimal values of reflectivity;
5. Value of a second derivative of a polynomial of a second degree approximated to the values in the λ_max_—λ_min_ interval at the average of wavelengths λ_max_ and λ_min_;
6. Standard deviation of a signal after detrending and subtracting the approximated polynomial;
7. The participation of high-frequency signals in signal energy after detrending and subtracting the approximated polynomial.

**Figure 2:**
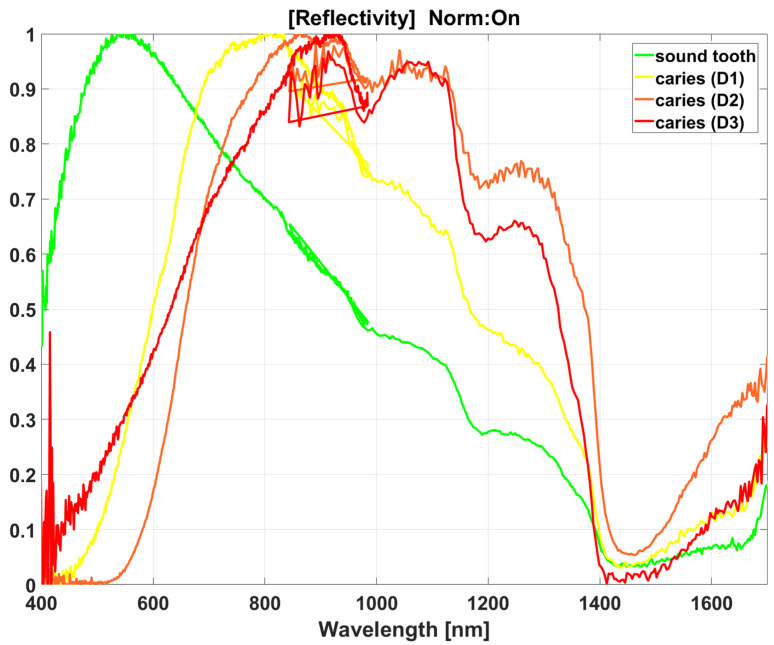
Normalized reflectivity curves representing sound tooth (green) and caries of extent D1 (yellow), D2 (orange), and D3 (red). Each reflectivity curve consists of signals from the two spectrometers, which overlap in the range of 845–984 nm. Noise was not filtered to prevent any alteration of the analyses.

ROIs were classified as sound (D0), demineralized within the outer half of the enamel (D1), demineralized within the inner half of the enamel (D2), and demineralized to the dentin (D3). Normalized reflectivity curves representative of each category are presented in Figure 2.

### 2.4. Histological Verification

Following the assessments, the radicular part of each tooth was embedded in a self-cured resin (Duracryl Plus; SpofaDental, Jicin, Czech Republic). The teeth were then cut into 150–200 μm thick sections through the ROIs using a low-speed precision saw (Isomet; Buehler, Esslingen, Germany) with water cooling. The direction of the cut was selected so that it did not interfere with other ROIs on the tooth. Each section was polished with 800-grit silicon carbide paper using EcoMet 30 (Buehler, Esslingen, Germany). The ROIs were then observed using the Leica DMLB microscope at 10× magnification, and their images were taken with the MC170 HD camera and LAS imaging software (all from Leica Microsystems, Wetzlar, Germany). The images were evaluated by an experienced histologist (T.K.) who was not familiar with the results of the previous assessments. The extent of caries was classified according to Downer’s criteria: 0 = no enamel demineralization or a narrow surface zone of opacity; 1 = enamel demineralization limited to the outer half of the enamel; 2 = demineralization involving the inner half of the enamel; 3 = demineralization involving the outer half of the dentin; 4 = demineralization involving the inner half of the dentin [44,45,46]. Table 3 presents the assumed correspondence of Downer’s criteria with other detection methods.

### 2.5. Statistical Analysis

The histological evaluation was adopted as a reference standard. The sensitivity, specificity, false positive rate, and false negative rate of each detection method were calculated for each lesion depth (D1–D3), as well as without differentiating lesion depth. The differences were considered significant if the 95% confidence intervals did not overlap. The agreement between the two investigators in ICDAS was evaluated using Cohen’s kappa and the intraclass correlation coefficient (ICC). In addition, the area under the ROC curve was calculated for each detection method. The statistical analyses were performed in RStudio (2022.12.0 Build 353; Boston, MA, USA).

## 3. Results

The histological evaluation revealed that 180 ROIs were sound, while 120 were carious (Downer’s D1–36, D2–52, D3–32, D4–0). Detailed results of each detection method stratified according to lesion depth are presented in Table 4.

In D1 lesions, there was no significant difference between visual inspection and the Diagnodent pen. However, they were both outperformed by DRS in all parameters except for sensitivity (Diagnodent pen) and false negative rate (visual inspection and Diagnodent pen). In D2 lesions, there were significant differences between the detection methods in sensitivity; the Diagnodent pen exhibited the lowest sensitivity, while the highest sensitivity was achieved with DRS. There was no significant difference between the methods in specificity and false negative rate but DRS had the highest accuracy, and its false positive rate was significantly lower than that of the Diagnodent pen. In D3 lesions, DRS resulted in the lowest sensitivity but the difference was significant only from the visual examination by the second investigator. In contrast, DRS significantly outperformed other methods in specificity and false positive rate. There was no significant difference between the methods in accuracy and false negative rate.

Table 5 presents the results without the differentiation of lesion depth. There was no significant difference between the detection methods in sensitivity and false negative rate but DRS outperformed other detection methods in specificity, accuracy, and false positive rate.

### 3.1. ICDAS

**Figure 3:**
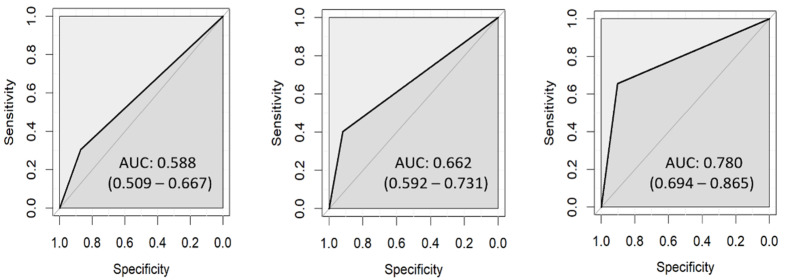
ROC curves for ICDAS assessed by L.H.: D1 (**left**), D2 (**center**), D3 (**right**).

**Figure 4:**
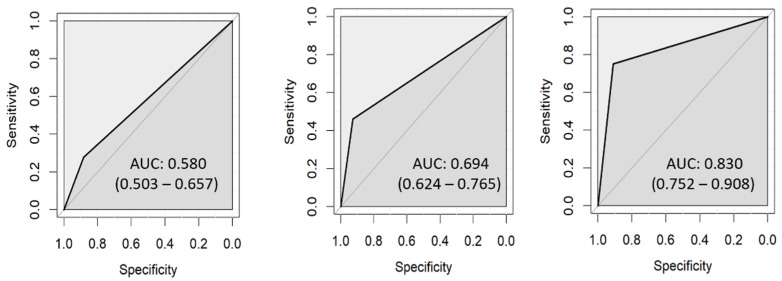
ROC curves for ICDAS assessed by J.C.: D1 (**left**), D2 (**center**), D3 (**right**).

The agreement between investigators was 80.7% (Cohen’s kappa 0.689, ICC 0.901). The first investigator (L.H.) identified 167 ROIs as sound but only 155 of them were correct. In carious lesions, 53 ROIs were correctly classified according to lesion depth (Table 6). The sensitivity increased with lesion depth: 31% for D1, 40% for D2, and 66% for D3. The ROC curves for each lesion depth are presented in Figure 3. The AUC was 0.588 (0.509–0.667) for D1, 0.662 (0.592–0.731) for D2, and 0.780 (0.694–0.865) for D3. The second investigator (J.C.) agreed with the histological evaluation in 157 sound and 58 carious ROIs (Table 7). His values of AUC were similar to the first examiner—0.580 (0.503–0.657) for D1, 0.694 (0.624–0.765) for D2, and 0.830 (0.752–0.908) for D3 (Figure 4).

### 3.2. Diagnodent Pen

**Figure 5:**
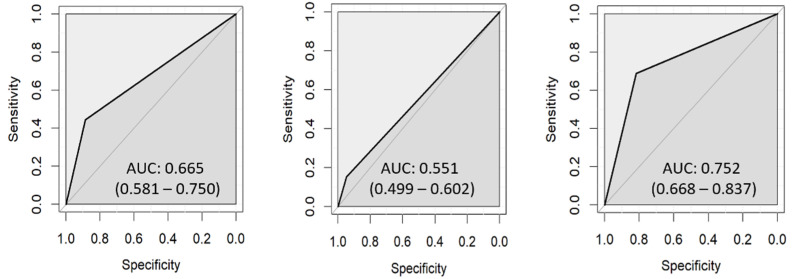
ROC curves for the Diagnodent pen: D1 (**left**), D2 (**center**), D3 (**right**).

Using the Diagnodent pen, 162 ROIs were identified as sound and 154 of them agreed with the histological classification. In carious lesions, 58 ROIs were correctly classified according to lesion depth (Table 8). Unlike visual inspection, the sensitivity of the Diagnodent pen did not increase with lesion depth, as the lowest value was recorded in D2 lesions (0.15). The AUC of the Diagnodent pen was 0.665 (0.581–0.750) for D1, 0.551 (0.499–0.602) for D2, and 0.752 (0.668–0.837) for D3 (Figure 5).

### 3.3. DRS

**Figure 6:**
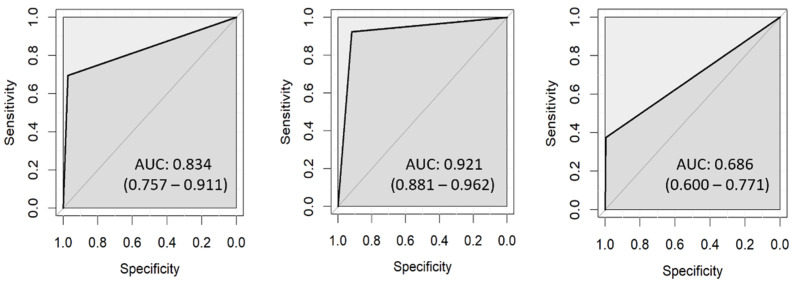
ROC curves for DRS: D1 (**left**), D2 (**center**), D3 (**right**).

DRS classified 187 ROIs as sound, which is markedly more compared with other detection methods. Out of the 187 ROIs, 176 were in agreement with the histological results. In carious lesions, 85 ROIs were correctly classified according to lesion depth (Table 9). However, DRS exhibited the lowest sensitivity for D3 caries (0.38) because 20 D3 lesions were classified as D2 (Table 9). Figure 6 presents the ROC curves for DRS and the AUC was 0.834 (0.757–0.911) for D1, 0.921 (0.881–0.962) for D2, and 0.686 (0.600–0.771) for D3.

## 4. Discussion

Early and accurate detection of dental caries plays an important role in dentistry, as it allows for caries arrest or minimally invasive treatment and therefore reduces the damage to hard dental tissues [47]. However, the detection of incipient caries is still challenging [48]. This in vitro study aimed to evaluate the performance of the recently introduced DRS and to compare it with other caries detection methods, i.e., visual detection using ICDAS and the Diagnodent pen. Histological evaluation served as the ground truth because clinical caries detection methods may fail to correctly identify the condition of hard dental tissues and therefore lead to misinterpretation [49,50,51,52]. The results showed that the performance of DRS in D1 and D2 caries was superior to visual inspection and the Diagnodent pen in almost all parameters, so the first null hypothesis was rejected. In D3 lesions, DRS exhibited the lowest sensitivity because it could not differentiate them from D2. In contrast, the accuracy of visual inspection and the Diagnodent pen tended to increase with lesion depth, and based on these results, the second null hypothesis was rejected as well.

Visual inspection of the tooth surface is a widely used method as it is fast and does not require any special equipment. It is commonly combined with probing because it increases the specificity of caries detection, however, only at the expense of decreased sensitivity [53]. Furthermore, as the explorer may damage hard dental tissues affected by incipient caries and transfer bacteria from the infected surfaces, some consider probing as obsolete [39] and it was therefore not used in this study. The outcomes of visual inspection published in the literature vary greatly due to its subjectivity and different methodological approaches. According to a meta-analysis published in 2015 [4], the pooled sensitivity and specificity of the visual detection of initial occlusal caries in permanent teeth in laboratory studies were 81.4% and 73.2%, respectively. In this study, the sensitivity and specificity of caries detection without the differentiation of caries extent (Table 5) was superior to the outcomes of the meta-analysis, 90% and 86–87%, respectively. The differentiation of caries extent resulted in a slightly higher specificity; on the other hand, sensitivity decreased because some carious lesions were incorrectly classified as smaller or larger. This could be partly caused by the fact that the ICDAS does not correspond well with Downer’s criteria (Table 3).

In some cases, such as hidden caries [18], a visual inspection may be insufficient and complementary caries detection methods such as laser fluorescence (Diagnodent pen) may be useful. Furthermore, the Diagnodent pen device provides numerical results, and it is, therefore, less dependent on the operator’s experience. In this study, without the differentiation of caries extent, the values of sensitivity (93%) and specificity (86%) were comparable with the literature data [54,55]. In D2 and D3 caries, the Diagnodent pen exhibited the highest false positive rate, primarily caused by the presence of stain or other surface contaminants [11,56], which limits the usefulness of the Diagnodent pen as a diagnostic tool [55].

A previous study showed that the Diagnodent pen readings were affected by the thickness of hard dental tissues present between the carious lesion and the instrument’s tip [57]. Consequently, the detection depth may be insufficient to detect occlusal caries [57]. While light emitted by the Diagnodent pen has a wavelength of 655 nm, DRS uses a broader range of wavelengths (400–1600 nm), which should allow for deeper penetration through hard dental tissues. However, the penetration depth also depends on the distance between the optical fibers—the larger the distance, the higher the detection depth [58,59,60]. In this study, the distance between the optical fibers was 0.85 mm, which seems to be suitable for the detection of demineralization within the enamel, as DRS outperformed the Diagnodent pen and visual inspection in D1 and D2 lesions. However, DRS struggled with differentiating D3 lesions from D2, which may be caused by the similarity of the reflectivity curves but could also suggest that the penetration depth of DRS was insufficient. In order to improve this result, it may be necessary to increase the distance between the optical fibers of the DRS probe. Nevertheless, even with the current settings, DRS exhibited the highest specificity and the lowest false positive rate in D3 lesions, showing promise as a caries detection method. This is further supported by the high sensitivity (91%) and specificity (98%) of DRS without distinguishing caries extent.

It may be seen as a limitation of this study that it was conducted on individual extracted teeth. This allowed for the ideal access to ROIs, which is important because a previous study showed that results were significantly affected by the inclination of the DRS probe [42]. Clinically, a perpendicular attachment of the probe to the tooth surface may be hard to achieve, especially on the proximal surfaces of the teeth. To improve the access, it would be helpful if the probe was bent and reduced in length. It can be also seen as a limitation that the correspondence between Downer’s criteria and the ICDAS and Diagnodent pen’s cut-off limits were not perfect, which could adversely affect the results with these methods.

## 5. Conclusions

In this in vitro study, the DRS prototype device exhibited superior performance than visual detection and the Diagnodent pen, particularly in the detection of incipient caries. In dentin caries, the sensitivity of the DRS device was limited, suggesting the need for its optimization. The performance of DRS also needs to be confirmed in clinical trials.

## Figures and Tables

**Table 1:** International Caries Detection and Assessment System (ICDAS).

Code	Description
0	Sound tooth surface: No evidence of caries after 5 s of air drying
1	First visual change in enamel: Opacity or discoloration (white or brown) is visible at the entrance to the pit or fissure seen after prolonged air drying
2	Distinct visual change in enamel visible when wet, lesion must be visible when dry
3	Localized enamel breakdown (without clinical visual signs of dentinal involvement) seen when wet and after prolonged drying
4	Underlying dark shadow from dentine
5	Distinct cavity with visible dentine
6	Extensive (more than half the surface) distinct cavity with visible dentine

According to Gugnani et al. [5].

**Table 2:** Diagnodent pen scale.

Cut-Off Limits	Clinical Lesion Depth
0–13	Sound
14–20	Enamel lesions
21–29	Caries in dentin–enamel junction
>29	Dentin caries

According to Hibst et al. [41].

**Table 3:** Assumed correspondence of Downer’s criteria with other detection methods.

Downer’s Criteria	ICDAS	Diagnodent Pen	DRS
0	Sound tooth surface	0–13 (Sound)	D0 (Sound)
1	First visual change in enamel	14–20 (Enamel caries)	D1 (Demineralization in the outer half of the enamel)
2	Distinct visual change in enamel	21–29 (Caries in dentin–enamel junction)	D2 (Demineralization in the inner half of the enamel)
3	Localized enamel breakdown	>29 (Dentin caries)	D3 (Demineralization of the dentin)

**Table 4:** Comparison of detection methods stratified according to lesion depth.

Caries Extent	Detection Method	Sensitivity (95% CI)	Specificity (95% CI)	Accuracy (95% CI)	False Negative (95% CI)	False Positive (95% CI)
D1	ICDAS (1)	0.31 (0.16–0.46)	0.87 (0.83–0.91)	0.80 (0.76–0.85)	0.10 (0.06–0.13)	0.76 (0.63–0.88)
	ICDAS (2)	0.28 (0.13–0.42)	0.88 (0.84–0.92)	0.81 (0.76–0.86)	0.10 (0.06–0.14)	0.76 (0.62–0.89)
	DIAGNOdent pen	0.44 (0.28–0.61)	0.89 (0.85–0.92)	0.83 (0.79–0.88)	0.08 (0.05–0.11)	0.65 (0.51–0.79)
	DRS	0.69 (0.54–0.84)	0.97 (0.95–0.99)	0.94 (0.91–0.97)	0.04 (0.02–0.06)	0.22 (0.08–0.36)
D2	ICDAS (1)	0.40 (0.27–0.54)	0.92 (0.89–0.95)	0.83 (0.78–0.88)	0.12 (0.08–0.16)	0.49 (0.33–0.64)
	ICDAS (2)	0.46 (0.33–0.60)	0.93 (0.90–0.96)	0.85 (0.80–0.89)	0.11 (0.07–0.15)	0.43 (0.28–0.58)
	DIAGNOdent pen	0.15 (0.06–0.25)	0.95 (0.92–0.98)	0.81 (0.76–0.86)	0.16 (0.11–0.20)	0.62 (0.41–0.83)
	DRS	0.92 (0.85–1.00)	0.92 (0.89–0.95)	0.92 (0.89–0.95)	0.02 (0.00–0.03)	0.29 (0.19–0.40)
D3	ICDAS (1)	0.66 (0.49–0.82)	0.90 (0.87–0.94)	0.88 (0.84–0.92)	0.04 (0.02–0.07)	0.55 (0.41–0.70)
	ICDAS (2)	0.75 (0.60–0.90)	0.91 (0.88–0.94)	0.89 (0.86–0.93)	0.03 (0.01–0.05)	0.50 (0.36–0.64)
	DIAGNOdent pen	0.69 (0.53–0.85)	0.82 (0.77–0.86)	0.80 (0.76–0.85)	0.04 (0.02–0.07)	0.69 (0.58–0.80)
	DRS	0.38 (0.21–0.54)	0.996 (0.99–1.00)	0.93 (0.90–0.96)	0.07 (0.04–0.10)	0.07 (0.00–0.38)

CI: Confidence interval.

**Table 5:** Comparison of detection methods without the differentiation of lesion depth.

Detection Method	Sensitivity (95% CI)	Specificity (95% CI)	Accuracy (95% CI)	False Negative (95% CI)	False Positive (95% CI)
ICDAS (1)	0.90 (0.83–0.95)	0.86 (0.80–0.91)	0.88 (0.83–0.91)	0.07 (0.04–0.13)	0.19 (0.13–0.27)
ICDAS (2)	0.90 (0.83–0.95)	0.87 (0.81–0.92)	0.88 (0.84–0.92)	0.07 (0.04–0.12)	0.18 (0.12–0.25)
DIAGNOdent pen	0.93 (0.87–0.97)	0.86 (0.80–0.90)	0.89 (0.85–0.92)	0.05 (0.02–0.10)	0.19 (0.13–0.27)
DRS	0.91 (0.84–0.95)	0.98 (0.94–0.99)	0.95 (0.92–0.97)	0.06 (0.03–0.11)	0.04 (0.01–0.09)

CI: Confidence interval.

**Table 6:** Comparison between ICDAS assessment by L.H. and histological classification.

ICDAS Code	Downer’s Class 0	Downer’s Class 1	Downer’s Class 2	Downer’s Class 3	Total
0	**155**	9	3	0	167
1	25	**11**	9	0	45
2	0	9	**21**	11	41
3	0	7	19	**21**	47
Total	180	36	52	32	300

Values in bold present the true positive results for each lesion depth.

**Table 7:** Comparison between ICDAS assessment by J.C. and histological classification.

ICDAS Code	Downer’s Class 0	Downer’s Class 1	Downer’s Class 2	Downer’s Class 3	Total
0	**157**	8	4	0	169
1	23	**10**	8	0	41
2	0	10	**24**	8	42
3	0	8	16	**24**	48
Total	180	36	52	32	300

Values in bold present the true positive results for each lesion depth.

**Table 8:** Comparison between the Diagnodent pen and histological classification.

Diagnodent Pen Value	Downer’s Class 0	Downer’s Class 1	Downer’s Class 2	Downer’s Class 3	Total
0–13	**154**	5	3	0	162
14–20	9	**16**	14	7	46
21–29	5	5	**8**	3	21
>29	12	10	27	**22**	71
Total	180	36	52	32	300

Values in bold present the true positive results for each lesion depth.

**Table 9:** Comparison between DRS pen and histological classification.

DRS Class	Downer’s Class 0	Downer’s Class 1	Downer’s Class 2	Downer’s Class 3	Total
D0	**176**	11	0	0	187
D1	4	**25**	3	0	32
D2	0	0	**48**	20	68
D3	0	0	1	**12**	13
Total	180	36	52	32	300

Values in bold present the true positive results for each lesion depth.

## Data Availability

The data presented in this study are available on reasonable request from the corresponding author.

## References

[1] Marinho V.C., Chong L.-Y., Worthington H.V., Walsh T. (2016). Fluoride mouthrinses for preventing dental caries in children and adolescents. Cochrane Database Syst. Rev..

[2] Horst J.A., Tanzer J.M., Milgrom P.M. (2018). Fluorides and Other Preventive Strategies for Tooth Decay. Dent. Clin. N. Am..

[3] Kocak N., Cengiz-Yanardag E. (2020). Clinical performance of clinical-visual examination, digital bitewing radiography, laser fluorescence, and near-infrared light transillumination for detection of non-cavitated proximal enamel and dentin caries. Lasers Med. Sci..

[4] Gimenez T., Piovesan C., Braga M.M., Raggio D.P., Deery C., Ricketts D.N., Ekstrand K., Mendes F.M. (2015). Visual Inspection for Caries Detection: A systematic review and meta-analysis. J. Dent. Res..

[5] Gugnani N., Pandit I.K., Srivastava N., Gupta M., Sharma M. (2011). International Caries Detection and Assessment System (ICDAS): A New Concept. Int. J. Clin. Pediatr. Dent..

[6] Dikmen B. (2015). ICDAS II criteria (international caries detection and assessment system). J. Istanb. Univ. Fac. Dent..

[7] Ekstrand K.R., Gimenez T., Ferreira F.R., Mendes F.M., Braga M.M. (2018). The International Caries Detection and Assessment System—ICDAS: A Systematic Review. Caries Res..

[8] Geibel M.-A., Carstens S., Braisch U., Rahman A., Herz M., Jablonski-Momeni A. (2017). Radiographic diagnosis of proximal caries—Influence of experience and gender of the dental staff. Clin. Oral Investig..

[9] Turchiello R.Z., Pedrotti D., Braga M.M., Rocha R.O., Rodrigues J.A., Lenzi T.L. (2019). Do undergraduate dental students perform well detecting and staging caries and assessing activity by visual examination? A systematic review and meta-analysis. Int. J. Paediatr. Dent..

[10] Todorova V., Filipov I., Petrova R. (2020). In Vitro Comparison of Several Methods for Initial Proximal Caries Detection. Folia Med..

[11] Pretty I.A. (2006). Caries detection and diagnosis: Novel technologies. J. Dent..

[12] Abogazalah N., Ando M. (2017). Alternative methods to visual and radiographic examinations for approximal caries detection. J. Oral Sci..

[13] Marinova-Takorova M., Anastasova R., Panov V.E. (2014). Comparative evaluation of the effectiveness of five methods for early diagnosis of occlusal caries lesions—In vitro study. J. IMAB—Annu. Proceeding Sci. Pap..

[14] Blazejewska A.I., Dacyna N., Niesiobędzki P., Trzaska M., Gozdowski D., Turska-Szybka A., Olczak-Kowalczyk D. (2016). Comparison of the detection of proximal caries in children and youth using DIAGNOcam and bitewing radiovisiography. Dent. Med. Probl..

[15] Alamoudi N., Khan J., El-Ashiry E., Felemban O., Bagher S., Al-Tuwirqi A. (2019). Accuracy of the DIAGNOcam and bitewing radiographs in the diagnosis of cavitated proximal carious lesions in primary molars. Niger. J. Clin. Pract..

[16] Gostanian H.V., Shey Z., Kasinathan C., Caceda J., Janal M.N. (2006). An in vitro evaluation of the effect of sealant characteristics on laser fluorescence for caries detection. Pediatr. Dent..

[17] Ekstrand K., Ricketts D.N.J., Kidd E.A.M. (1997). Reproducibility and Accuracy of Three Methods for Assessment of Demineralization Depth on the Occlusal Surface: An in vitro Examination. Caries Res..

[18] Ricketts D., Kidd E., Weerheijm K., De Soet H. (1997). Hidden caries: What is it? Does it exist? Does it matter?. Int. Dent. J..

[19] Takamori K., Hokari N., Okumura Y., Watanabe S. (2001). Detection of Occlusal Caries under Sealants by Use of a Laser Fluorescence System. J. Clin. Laser Med. Surg..

[20] Heinrich-Weltzien R., Weerheijm K.L., Kühnisch J., Oehme T., Stösser L. (2002). Clinical evaluation of visual, radiographic, and laser fluorescence methods for detection of occlusal caries. ASDC J. Dent. Child..

[21] Anttonen V., Seppä L., Hausen H. (2003). Clinical Study of the Use of the Laser Fluorescence Device DIAGNOdent for Detection of Occlusal Caries in Children. Caries Res..

[22] Lussi A., Hellwig E. (2006). Performance of a new laser fluorescence device for the detection of occlusal caries in vitro. J. Dent..

[23] Rodrigues J., Hug I., Diniz M., Lussi A. (2008). Performance of Fluorescence Methods, Radiographic Examination and ICDAS II on Occlusal Surfaces in vitro. Caries Res..

[24] Alkurt M.T., Peker I., Arisu H.D., Bala O., Altunkaynak B. (2007). In vivo comparison of laser fluorescence measurements with conventional methods for occlusal caries detection. Lasers Med. Sci..

[25] Tassoker M., Ozcan S., Karabekiroglu S. (2020). Occlusal Caries Detection and Diagnosis Using Visual ICDAS Criteria, Laser Fluorescence Measurements, and Near-Infrared Light Transillumination Images. Med. Princ. Pract..

[26] Pourhashemi S., Jafari A., Motahhari P., Panjnoosh M., Fard M.K., Sanati I., Sahadfar M., Pariab M. (2009). An *in-vitro* comparison of visual inspection, bite-wing radiography, and laser fluorescence methods for the diagnosis of occlusal caries. J. Indian Soc. Pedod. Prev. Dent..

[27] Nokhbatolfoghahaie H., AliKhasi M., Chiniforush N., Khoei F., Safavi N., Zadeh B.Y. (2013). Evaluation of Accuracy of DIAGNOdent in Diagnosis of Primary and Secondary Caries in Comparison to Conventional Methods. J. Lasers Med. Sci..

[28] Kockanat A., Unal M. (2017). In vivo and in vitro comparison of ICDAS II, DIAGNOdent pen, CarieScan PRO and SoproLife camera for occlusal caries detection in primary molar teeth. Eur. J. Paediatr. Dent..

[29] Castilho L.S., Cotta F.V., Bueno A.C., Moreira A.N., Ferreira E.F., Magalhães C.S. (2016). Validation of DIAGNOdent laser fluorescence and the International Caries Detection and Assessment System (ICDAS) in diagnosis of occlusal caries in permanent teeth: An in vivo study. Eur. J. Oral Sci..

[30] Hill W., Petrou V. (2000). Caries Detection by Diode Laser Raman Spectroscopy. Appl. Spectrosc..

[31] Samek O., Telle H.H., Beddows D.C. (2001). Laser-induced breakdown spectroscopy: A tool for real-time, in vitro and in vivo identification of carious teeth. BMC Oral Health.

[32] Lussi A., Hibst R., Paulus R. (2004). DIAGNOdent: An Optical Method for Caries Detection. J. Dent. Res..

[33] Izawa T., Wakaki M. Application of Laser Raman Spectroscopy to Dental Diagnosis. Proceedings of the SPIE BiOS.

[34] Liu L., Tang J., Li S.Z. A new method for caries diagnosis by characteristic spectrum. Proceedings of the 2009 2nd Inter-national Conference on Biomedical Engineering and Informatics.

[35] Ruohonen M., Palo K., Alander J. (2013). Spectroscopic Detection of Caries Lesions. J. Med. Eng..

[36] Procházka A., Charvát J., Vyšata O., Mandic D. (2022). Incremental deep learning for reflectivity data recognition in stomatology. Neural Comput. Appl..

[37] Charvát J., Procházka A., Fričl M., Vyšata O., Himmlová L. (2020). Diffuse reflectance spectroscopy in dental caries detection and classification. Signal Image Video Process..

[38] Schwarz R.A., Gao W., Daye D., Williams M.D., Richards-Kortum R., Gillenwater A. (2008). Autofluorescence and diffuse reflectance spectroscopy of oral epithelial tissue using a depth-sensitive fiber-optic probe. Appl. Opt..

[39] Lussi A., Francescut P. (2003). Performance of Conventional and New Methods for the Detection of Occlusal Caries in Deciduous Teeth. Caries Res..

[40] Luczaj-Cepowicz E., Marczuk-Kolada G., Obidzinska M., Sidun J. (2019). Diagnostic validity of the use of ICDAS II and DIAGNOdent pen verified by micro-computed tomography for the detection of occlusal caries lesions—An in vitro evaluation. Lasers Med. Sci..

[41] Hibst R., Paulus R., Lussi A. (2001). Detection of Occlusal Caries by Laser Fluorescence: Basic and Clinical Investigations. Med. Laser Appl..

[42] Fričl M. (2020). Analysis of Spectroscopic Images of Hard Dental Tissues.

[43] Martynek D. (2022). Diffuse Reflectance Spectroscopy in Dental Tissue Analysis.

[44] Downer M.C. (1975). Concurrent Validity of an Epidemiological Diagnostic System for Caries with the Histological Appearance of Extracted Teeth as Validating Criterion. Caries Res..

[45] Attrill D.C., Ashley P. (2001). Occlusal caries detection in primary teeth: A comparison of DIAGNOdent with conventional methods. Br. Dent. J..

[46] Subka S., Rodd H., Nugent Z., Deery C. (2019). In vivo validity of proximal caries detection in primary teeth, with histological validation. Int. J. Paediatr. Dent..

[47] Huth K., Neuhaus K., Gygax M., Bücher K., Crispin A., Paschos E., Hickel R., Lussi A. (2008). Clinical performance of a new laser fluorescence device for detection of occlusal caries lesions in permanent molars. J. Dent..

[48] Bengtson A.L., Gomes A.C., Mendes F.M., Cichello L.R., Bengtson N.G., Pinheiro S.L. (2005). Influence of examiner’s clinical experience in detecting occlusal caries lesions in primary teeth. Pediatr. Dent..

[49] Reis A., Mendes F.M., Angnes V., Angnes G., Grande R.H.M., Loguercio A.D. (2006). Performance of methods of occlusal caries detection in permanent teeth under clinical and laboratory conditions. J. Dent..

[50] Jablonski-Momeni A., Stachniss V., Ricketts D., Heinzel-Gutenbrunner M., Pieper K. (2008). Reproducibility and Accuracy of the ICDAS-II for Detection of Occlusal Caries in vitro. Caries Res..

[51] Mitropoulos P., Rahiotis C., Stamatakis H., Kakaboura A. (2010). Diagnostic performance of the visual caries classification system ICDAS II versus radiography and micro-computed tomography for proximal caries detection: An in vitro study. J. Dent..

[52] Soviero V., Leal S., Silva R., Azevedo R. (2012). Validity of MicroCT for in vitro detection of proximal carious lesions in primary molars. J. Dent..

[53] Bader J.D., Shugars D.A., Bonito A.J. (2001). Systematic Reviews of Selected Dental Caries Diagnostic and Management Methods. J. Dent. Educ..

[54] Foros P., Oikonomou E., Koletsi D., Rahiotis C. (2021). Detection Methods for Early Caries Diagnosis: A Systematic Review and Meta-Analysis. Caries Res..

[55] Bader J.D., Shugars D.A. (2004). A systematic review of the performance of a laser fluorescence device for detecting caries. J. Am. Dent. Assoc..

[56] Walsh L.J. (2018). Caries Diagnosis Aided by Fluorescence. Dental Caries.

[57] Markowitz K., Stenvall R., Graye M. (2012). The Effect of Distance and Tooth Structure on Laser Fluorescence Caries Detection. Oper. Dent..

[58] Shoaib Z., Kamran M.A., Mannan M.M.N., Jeong M.Y. (2019). Approach to optimize 3-dimensional brain functional activation image with high resolution: A study on functional near-infrared spectroscopy. Biomed. Opt. Express.

[59] Ohnishi M., Kusakawa N., Masaki S., Honda K., Shimada Y., Fujimoto I., Hirao K. (2003). Investigation on deep layer meas-urements in the cerebral cortex within the adult head by near infrared spectroscopy using an absorbance difference technique. J. Near Infrared Spectrosc..

[60] Si W., Xiong J., Huang Y., Jiang X., Hu D. (2022). Quality Assessment of Fruits and Vegetables Based on Spatially Resolved Spectroscopy: A Review. Foods.

